# Retraction: The effect of autophagy and mitochondrial fission on Harderian gland is greater than apoptosis in male hamsters during different photoperiods

**DOI:** 10.1371/journal.pone.0281852

**Published:** 2023-02-10

**Authors:** The PLOS ONE Editors

Following the publication of this article [1], the first author requested correction of images in Figs 3 and 5. Following editorial assessment, additional concerns were raised regarding similarity to a previous study from the same group [2], the results presented in multiple figures and animal welfare standards. Specifically,

- There appears to be a large amount of text overlap between this article [1] and another article with common authors [2], and the two studies have a similar study design and report the same ethics approval number.
- The following images in [1] and [2] appear to fully or partially overlap despite representing different conditions:
  ■ SP panels (DAPI, AF647) in Fig 5 of [1] and MP panels (DAPI, AF647) in Fig 4 of [2].
  ■ β-actin in Fig 10A of [1] and β-actin in Fig 7A of [2] with exposure and/or contrast altered.
  ■ The first panel in S1 File of [1] and the β-actin panel in Fig 6 of [2].
- The following images within [1] appear to fully or partially overlap despite representing different conditions:
  ■ SP and LP panels in Fig 3.
  ■ AANAT panel in Fig 7A and CS panel in Fig 10A.
  ■ HIOMT panel in Fig 7A and bcl2 panel in Fig 8A.
- During discussions, the first author stated that different methods of standardisation were used for earlier and later western blots, but they were unable to say which blots were analysed by which method. They provided a data table in which many β-actin values appeared to be repeated and stated that one β-actin was used to standardize multiple target proteins of the same sample.
- According to an ethics approval document provided in follow-up discussions, ether was used for anesthesia without a specific scientific justification for its use which questions compliance of this article with *PLOS ONE*’s submission guidelines. This is inconsistent with the article which states that carbon dioxide was used for euthanasia.
- According to the methods section [1], wild hamsters were caught for this research without a scientific justification for doing so, which questions compliance with PLOS policy on animal research and introduces concerns about the reliability of the findings.

These concerns were discussed with the first author. They stated that all the data underlying the results in this article remain available, except for the original uncropped image for the β-actin panel in Fig 9A.

The first author clarified that the two articles [1, 2] report data from the same study carried out using male and female hamsters, respectively. The editors consider that the related article [2] should have been highlighted at the time of submission, in line with ethical publishing practices.

The first author acknowledged that the overlap between figures were caused by errors in file storage. They provided updated versions of these figures in which the following panels were replaced: SP panel in Fig 3, all panels in Fig 7A, SP panels in Fig 5, β-actin in Fig 10A

In relation to western blot issues, information and replacement data tables provided during discussions did not resolve the concerns.

In light of the above concerns which question the reliability of the data, and compliance with animal research policies, the *PLOS ONE* Editors retract this article.

JHX, XYZ, XCG, MW, HLX, LC, and LXX agreed with the retraction. JHX and MW stand by the article’s findings. ZW, and JJM either did not respond directly or could not be reached.
